# Gaze Orienting in the Social World: An Exploration of the Role Played by Caregiving Vocal and Tactile Behaviors in Infants with Visual Impairment and in Sighted Controls

**DOI:** 10.3390/brainsci14050474

**Published:** 2024-05-08

**Authors:** Serena Grumi, Elena Capelli, Federica Morelli, Luisa Vercellino, Eleonora Mascherpa, Chiara Ghiberti, Laura Carraro, Sabrina Signorini, Livio Provenzi

**Affiliations:** 1 Developmental Psychobiology Lab, IRCCS Mondino Foundation, 27100 Pavia, Italy; serena.grumi@mondino.it (S.G.); luisa.vercellino@mondino.it (L.V.); 2 Department of Brain and Behavioral Sciences, University of Pavia, 27100 Pavia, Italy; elena.capelli@unipv.it (E.C.); chiara.ghiberti01@universitadipavia.it (C.G.); laura.carraro02@universitadipavia.it (L.C.); 3 Developmental Neuro-Ophthalmology Unit, IRCCS Mondino Foundation, 27100 Pavia, Italy; federica.morelli@mondino.it (F.M.); sabrina.signorini@mondino.it (S.S.)

**Keywords:** attention, infants, parent, sensory processing, still face, stress, visual impairment

## Abstract

Infant attention is a cognitive function that underlines sensory–motor integration processes at the interface between the baby and the surrounding physical and socio-relational environment, mainly with the caregivers. The investigation of the role of non-visual inputs (i.e., vocal and tactile) provided by the caregivers in shaping infants’ attention in the context of visual impairment is relevant from both a theoretical and clinical point of view. This study investigated the social attention (i.e., gaze orientation) skills in a group of visually impaired (VI) and age-matched sighted controls (SCs) between 9 and 12 months of age. Moreover, the role of VI severity and maternal vocalizations and touch in shaping the social attention were investigated. Overall, 45 infants and their mothers participated in a video-recorded 4 min interaction procedure, including a play and a still-face episode. The infants’ gaze orientation (i.e., mother-directed, object-directed, or unfocused) and the types of maternal vocalizations and touch (i.e., socio-cognitive, affective) were micro-analytically coded. Maternal vocalizations and touch were found to influence gaze orientation differently in VI infants compared SCs. Moreover, the group comparisons during the play episode showed that controls were predominantly oriented to the mothers, while VI infants were less socially oriented. Visual impairment severity did not emerge as linked with social attention. These findings contribute to our understanding of socio-cognitive developmental trajectories in VI infants and highlight the need for tailored interventions to promote optimal outcomes for VI populations.

## 1. Introduction

Infant attention is a cognitive function that underlines sensory–motor integration processes at the interface between the baby and the surrounding physical and socio-relational environment [1,2]. When infants engage in interactions with their caregivers to explore the social environment, their attention and sensory–motor integration processes can be influenced by various types of stimuli, including visual, auditory, and tactile stimulation. Infant research thus far has primarily focused on examining the pivotal role of visual stimuli in scaffolding infants’ attention and in steering and promoting the emergence of adaptive social behaviors towards interactive partners [3,4]. For instance, studies investigating patterns of selective attention have largely confirmed infants’ early interest in human faces [5,6] and the early display of contingent responses to subtle changes in the emotional expression of adult conspecifics [7,8]. More recently, there has been a growing interest in exploring the role of non-visual stimuli in shaping infants’ attention in early interactive exchanges with their caregivers [9,10].

From the intrauterine environment onward, infants are exposed to human voices, particularly that of the mother, as sounds and vibrations are transmitted to the developing auditory mechanisms of the fetus [11]. The distinct impact of maternal vs. unknown adults’ voices on infants’ attention has been largely evidenced from the early weeks of life, affecting sucking behaviors [12], as well as the growth and functioning of the cerebral cortex and the autonomic nervous system [11,13] and eliciting sensible heart-rate responses [14]. Also, the types of parental verbalizations may play a specific role, for example, Ferrari et al. [15] showed that fetuses at 25 weeks of gestation were selectively responsive to specific maternal vocalizations, exhibiting a congruent change in their mouth motor behaviors. This suggests that the sensorimotor mechanisms involved in auditory and motor processing are precociously coordinated.

Infants’ attention and cognitive development can also be shaped by tactile stimulations, like early social and affectionate touch provided by the caregivers [16,17] Infants exhibit a preference for gentle tactile stimulation at low speeds, such as maternal affectionate touch and caresses, which activate the C-tactile fibers [18,19]. These fibers play a crucial role in processing socio-affective touch and are active and responsive from birth, apparently even in preterm infants [17,20,21,22]. Multisensory stimulation incorporating social touch of this nature could potentially aid early learning processes. For instance, four-month-old infants habituated to a typically uninteresting individual with an averted gaze demonstrated the ability to recognize and discriminate such stimuli when parents provided low-speed stroking [16]. In contrast, exposure to the task without tactile support did not yield the same recognition or discrimination.

It is possible to hypothesize that it is the mix of visual, vocal, and tactile stimulations provided by the caregivers favors and supports infants’ sensory integration, leading to trustworthy expectations about the surrounding environment and promoting early social attention skills [23,24,25]. However, a question arises regarding the impact of disrupted sensory channels, such as in the case of blind and visually impaired (VI) children. The absence or restriction of visual input during the development of brain networks associated with social attention could potentially result in a delay in socio-cognitive development [26]. This poses the necessity to further explore the role of non-visual inputs to obtain the necessary basis of evidence to develop early interventions that can maximize the potential for sensory integration, even in case, of less-than-optimal neuro-visual development [27].

As outlined in a recent systematic review, there has been a lack of comprehensive study on the socio-cognitive developmental trajectories of visually impaired (VI) children. Indeed existing literature reports on their interactive behaviors are frequently outdated, rely on small samples, suffer from methodological constraints, and typically yield inconclusive findings [28]. The majority of previous studies reported that blind and VI infants exhibit decreased responsiveness to social cues and attempts by caregivers to engage their attention [29,30]: 2-year-old children did not exhibit consistent reactions to their mother’s voice or touch, as indexed by fewer smiles and vocalizations when compared with sighted controls [31,32,33].

Importantly, a recent study suggested that the visual acuity of 1-year-old VI children accounted for the largest amount of variance in joint engagement. This underscores the importance of considering residual vision as a critical factor when examining socio-cognitive interaction skills in VI populations [34]. Furthermore, the stimulation received by visually impaired infants may also be influenced by parents’ challenges in adjusting to the informational requirements of their infants during play interactions [28,35]. Visual impairment can impact both the amount and substance of parental verbal interactions. Mothers of VI children aged 9 months to 3.5 years old engaged in fewer verbal interactions when compared to counterparts of sighted controls, potentially because their children often did not display clear responsiveness to parental initiatives, such as pointing or reaching out to express interest [36]. Additionally, Moore and McConachie [37] observed that mothers conversed less with 18-month-old children who had limited or no vision, offering fewer verbal labels and descriptions. Significantly, these challenges in interaction experienced by parents may further intensify their emotional strain, resulting in elevated levels of parenting stress, depression, and anxiety [38,39]. Consequently, this emotional burden could adversely impact the quality of stimulation provided to the child [40].

To date, no study has specifically investigated how different types of parental verbal and touch stimulation may modulate attention orientation in visually impaired (VI) infants. Addressing this literature gap holds promise for providing critical insights for both theoretical understanding and clinical advancement. First, it could represent a step beyond the primacy of the visual channel in a large part of the infant research tradition. Second, understanding the specific interactive challenges associated with visual impairment and exploring the potential utilization of alternative sensory modalities would not only support parenting in such circumstances but also identify new targets for rehabilitation aimed at enhancing socio-cognitive and attentional skills in children with VI.

## 2. Aims

The present study generally aims to deepen the knowledge on social attention skills, considered a product of early sensory–motor integration, in VI infants by investigating how different types of parental verbal and touch stimulation may modulate the attention orientation in VI infants. The specific aims of the study were (1) to assess during a playful interaction the association of maternal caregiving behaviors (i.e., vocalizations and touch) with social attention (i.e., gaze orientation) in VI and age-matched sighted controls (SCs) between 9 and 12 months of age; (2) to investigate differences in social attention between the two groups of children (i.e., VI and SCs) during playful and no-interaction (still face) episodes; and (3) to assess the impact of visual impairment severity on children’s social attention.

## 3. Materials and Methods

### 3.1. Participants

Overall, 45 infants and their mothers participated in the study: 24 visually impaired (VI) and 21 sighted infants (SIs). The groups were matched for sex (VI: 18 males, 63%; SIs: 14 males, 66% X^2^(1) = 0.38, *p* = 0.538) and age at testing (VI: M = 11.2 months, SD = 1.08; SIs: M = 11.1, SD = 1.06; t(43) = −0.19, *p* = 0.852).

Infants in the SC group were considered eligible for the study if (1) they had no history of visual impairments or other sensory or neurological conditions and (2) they were born at term (>37 gestational weeks) after healthy pregnancies. Infants in the VI group were eligible if they had received a clinical diagnosis of low vision based on standardized tests (i.e., Teller acuity cards) [41] and did not meet the criteria for blindness (i.e., they perceived at least the low-vision card). For infants born preterm (n = 5), corrected age was considered. Visual impairment could be of peripheral, cerebral, or oculomotor origin if it resulted in reduced grating acuity. Peripheral visual impairment (PVI) is due to the involvement of the pre-geniculostriate primary visual pathways (i.e., eye and optic nerve, as in inherited retinal dystrophy or congenital cataract), while cerebral visual impairment (CVI) is due to the involvement of retro-geniculostriate visual pathways, and ocular motor impairment (OMI) refers to abnormalities in ocular motility (fixation, smooth pursuit, saccades) that can cause reduced grating acuity. In our cohort, all infants but 4 (3 diagnosed with isolated congenital cataract and 1 diagnosed with albinism) underwent brain MRI that showed abnormalities in 8 (slight, non-specific alterations due to metabolic errors in 2 infants with macular dystrophy; lesions consistent with distress during delivery or periventricular leukomalacia due to prematurity in 5 infants with CVI; slight polymicrogyria in 1 infant with CVI). As for ethology, 7 infants presented CVI related to central issues (e.g., central nervous system lesions or malformations), 9 presented PVI (e.g., congenital cataract; retinopathy or eye malformations), and 8 presented infantile nystagmus syndrome. Concerning the degree of VI, 15 (63%) infants presented severe low vision and 9 (37%) moderate low vision [41].

### 3.2. Procedures and Measures

Mother–child dyads were consecutively enrolled at the Developmental Neuro-Ophthalmology Unit of the IRCCS Mondino Foundation (Pavia) according to the inclusion criteria described in the Participants section. All parents provided written informed consent to participate. The study was approved by the Ethics Committee of Pavia (Prot. 20200038007, 20 April 2020).

To investigate how infants modulate attention in the presence and absence of maternal vocal and tactile stimuli, we adopted two of the three episodes of the well-acknowledged face-to-face still-face (FFSF) paradigm, a widely used observational procedure to study socio-emotional and socio-cognitive behavior in typically developing [42,43] and at-risk infants [25,44]. We followed procedures validated in previous research ([43]). Mothers and infants participated in a video-recorded interaction. Infants were seated in a highchair with the mothers seated in front of them at a comfortable distance (approximately 40 cm). Two cameras were used, one focused on the infant and the other focused on the mother. The FFSF paradigm comprised three episodes: (1) a 2 min face-to-face play interaction (play); (2) a 2 min period of experimental manipulation of maternal responsiveness during which mothers were instructed to avoid any gestural, vocal, tactile communication with the infant, keeping eye contact and a still, poker face expression (still face); and (3) a 2 min face-to-face play interaction (reunion). For the purposes of this study, only the first two episodes were analyzed. For the play episode, mothers were instructed to play as they usually did at home, while during the still-face episode, mothers were instructed to stop the interaction and maintain a neutral expression while looking at their infants. For coding purposes, the videos from the two cameras were edited off-line to produce a single video with simultaneous frontal view of the face, hands, and torso of infant and mother.

Videos were micro-analytically coded (in 2 s bins) for the infant’s gaze orientation and mother’s vocal production and touch behavior. Continuous measures of percentage of time were computed for each measure. The infants’ gaze orientation (GO) was coded as: (a) maternal gaze orientation (i.e., the infant’s attentional focus is on the interactive partner); (b) object-directed gaze (i.e., the infant is exploring or visually scanning an object); or (c) unfocused (i.e., infant’s attentional focus is not directed towards an object or the interactive partner and no eye movements are present suggesting that the infant is engaged in active visual scanning or exploration of the environment). Maternal vocal productions were coded as (a) socio-cognitive (i.e., verbalizations that included requests, explanations, attempts to get the attention of the infant, and comments related to the infant’s mental world) and (b) nurturing (i.e., verbalizations that included playful vocal productions like singing, laughing, nursery rhymes, or imitations of previous vocal production of the infant, affectionate comments, and soothing speech). Maternal touch behaviors were categorized as (a) pragmatic, i.e., static touch, touch with a functional not interactive aim (e.g., supporting the posture or moving the body of the infant), or a cognitive function (e.g., getting the attention of the infant) and (b) nurturing, i.e., playful (e.g., tickle, shake, squeeze) or affectionate touches (e.g., kissing, stroking, or massaging).

Mothers filled in three validated questionnaires assessing emotional mental health: (a) the Beck Depression Inventory, BDI [45], (b) the State subscale of the State-Trait Anxiety Inventory, STAI [46], and (c) the Parenting Stress Index—short form, PSI-SF [47]. The BDI-II Italian adaptation [48] is a 21-item questionnaire providing a descriptive, non-diagnostic account of depressive symptoms’ severity. Items are rated on a 4-point Likert scale with a total continuous score ranging from 0 (low) to 63 (high). The Italian adaptation of the State Anxiety subscale of the STAI-Y [49] is a 20-item questionnaire providing a descriptive, non-diagnostic account of anxiety symptoms’ severity. Items are rated on a 4-point Likert scale with a total continuous score ranging from 20 (low) to 80 (high). The PSI-SF is a 36-item questionnaire that investigates stress in the parent and child systems and measures stressors related to child characteristics, parental characteristics, and situational and demographic factors. Items are rated on a 5-point Likert scale with a total continuous score ranging from 36 (low) to 180 (high).

## 4. Results

### 4.1. Sample Description

The groups of infants (VI and SIs) were matched for sex (VI: 18 males, 63%; SIs: 14 males, 66%, X^2^(1) = 0.38, *p* = 0.538) and age at testing (VI: mean = 11.2 months, SD = 1.08; SIs: mean = 11.1, SD = 1.06, t(43) = −0.19, *p* = 0.852). Furthermore, no statistically significant difference emerged in measures of maternal depressive symptoms and interactive behaviors in the play episode (see Table 1).

### 4.2. Associations between Maternal Behaviors and Infants’ Social Attention

Correlation statistics (Spearman’s rho) between maternal behaviors and infants’ gaze in the play episode for the two groups are presented in Table 2. In the SC group, maternal pragmatic touch was positively associated with unfocused GO, socio-cognitive vocal production was positively associated with unfocused GO and negatively associated with maternal GO, and nurturing vocal production was positively associated with maternal GO. Conversely, in the group of VI infants, maternal pragmatic touch was positively associated with object GO and negatively associated with maternal GO, and nurturing vocal production was positively associated with unfocused GO and negatively associated with object GO.

### 4.3. Differences in Social Attention between VI and SC Children

**Figure 1:**
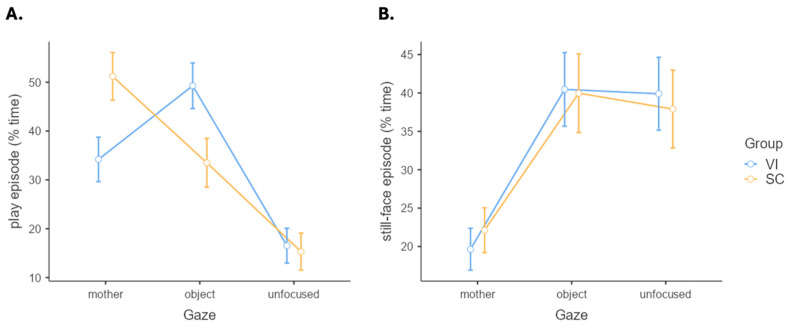
Gaze orientation (GO) for the two groups in the play (**A**) and the still-face (**B**) episodes. Note. VI, visual impairment; SC, sighted control; whiskers represent standard error.

Two separate analyses of variance (ANOVAs) were performed to compare the GO (mother-directed, object-directed, and unfocused GO) of the two groups of children (VI and SCs) during the play episode (Figure 1A) and the still episode (Figure 1B).

In the play episode, a significant effect of GO emerged (*F*(2, 86) = 15.39, *p* < 0.001), with no significant group effect (*F*(1, 43) < 0.001, *p* > 0.999). The simple effects were further qualified by a significant interaction effect (*F*(2, 86) = 4.55, *p* = 0.013). Post hoc paired comparisons showed higher maternal GO in SCs compared to VI infants, *t*(43) = 17.00, *p* = 0.014 and higher object GO in VI infants compared to SCs (*t*(43) = −15.78, *p* = 0.026). In the still-face episode, a significant GO effect emerged (*F*(2, 86) = 8.23, *p* < 0.001), while group effect (*F*(1, 43) < 0.01, *p* > 0.999) and the interaction term (*F*(2, 86) = 0.09, *p* = 0.913) were not significant. Post hoc paired comparisons showed that in both groups, infants displayed lower maternal GO compared to object GO (*t*(43) = −4.27, *p* < 0.001) and unfocused GO (*t*(43) = −4.04, *p* < 0.001).

### 4.4. Visual Impairment Severity and Social Attention

**Figure 2:**
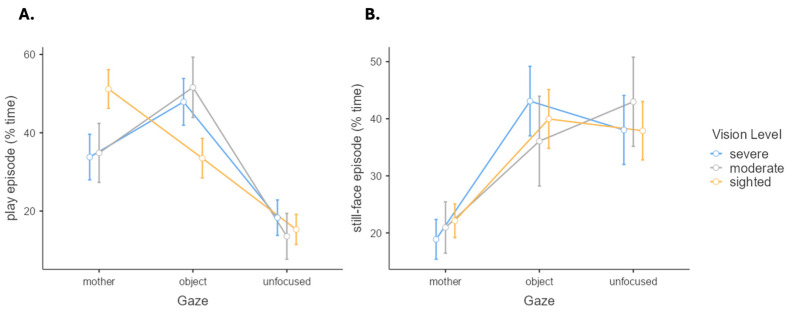
Gaze orientation (GO) for the three severity subgroups in the play (**A**) and the still-face (**B**) episodes. Note. Whiskers represent standard error.

Two separate analyses of variance (ANOVA) were performed to compare the GO (mother-directed, object-directed, and unfocused GO) among three groups of children with different vision levels (i.e., severe, moderate, and sighted) during the play episode (Figure 2A) and the still-face episode (Figure 2B). GO data for the three visual impairment severity subgroups are plotted in Figure 2. In the play episode, a significant GO effect emerged (*F*(2, 86) = 14.00, *p* < 0.001), while the group effect was non-significant (*F*(1, 43) < 0.001, *p* > 0.999). The interaction effect tended toward statistical significance (*F*(2, 86) = 2.31, *p* = 0.065). In the still-face episode, a significant effect of GO emerged (*F*(2, 86) = 7.48, *p* = 0.001), whereas no significant effects emerged for the group (*F*(1, 43) < 0.01, *p* > 0.999) or the interaction term (*F*(2, 86) = 0.22, *p* = 0.929).

## 5. Discussion

Infants’ attention and sensory–motor integration processes are fundamental components of early cognitive development, providing a foundation for interaction with the environment and caregivers. Recent studies have sought to expand beyond the dominance of the visual channel, emphasizing the role of auditory and tactile stimuli in shaping infants’ attention. This study aimed to address the gap in the literature regarding social attention skills in infants with visual impairment by assessing the influence of maternal non-visual stimuli (i.e., cognitive/pragmatical or nurturing vocalizations and touch) on infants’ attention orientation and comparing attention orientation in two groups of sighted and visually impaired infants across a well-validated behavioral paradigm.

Differently from what was shown in previous research [50], the mothers of our VI and SC groups did not differ in the vocal or touch stimuli they exhibited during the interaction. Nonetheless, our result suggested that these interactive caregivers’ behaviors shaped infants’ social attention behaviors, consistent with the available literature [15,16]. However, interestingly, maternal caregiving behaviors, such as pragmatic touch and socio-cognitive vocalizations, were found to be associated with different patterns of infant gaze orientation in infants with visual impairment compared to sighted counterparts. Indeed, nurturing verbalizations were the most helpful stimuli for maternal gaze orientation for sighted infants, while they were associated with lack of orientation in infants with visual impairment. For this latter group, no specific caregiving behaviors emerged as being associated with the social orientation of attention, while pragmatic touch appeared to improve infants’ attention orientation. These results are mainly explorative, considering that little is known about the maternal touch and verbal scaffolding to the attention orientation specifically in the presence of visual impairment [50,51]. However, they contribute to underscoring the need for tailored interventions to support socio-cognitive development in infants with visual impairment, as highlighted in previous literature [26,27].

The group comparisons during the play episode showed that controls were predominantly oriented to the mothers, while infants with visual impairment were less socially oriented. These findings are consistent with a recent review of the literature on the characterization of parent–infant interaction in the presence of visual impairment [28]. For example, Rogers and Puchalski [32] reported that blind and partially sighted children during the first and second year of life tended to be less responsive to their mother’s communicative signals for longer periods in comparison to sighted counterparts. More recently, Nagayoshi and colleagues [31] investigating 18 dyads of one-year-old visually impaired infants found that they tended not to turn their faces or eyes toward their mothers in response to maternal verbalizations. Interestingly, in the still-face condition, both groups showed similar attention orientation patterns, suggesting similar outputs in the absence of external stimulations. On an exploratory note, the severity of visual impairment did not influence social attention in our sample of visually impairment: gaze orientation in the subgroups characterized by moderate and severe visual impairment reflected similar patterns in both play and still-face episodes.

The present study has some limitations. First, given the clinical characteristics of the investigated infant population and the consequent enrollment challenges, the sample size is relatively low, with a consequent impact on the analysis’s statistical power. Second, as presented in the sample description, there is variability in the infants’ diagnosis and in their levels of impairment. The impact of visual impairment level has been controlled for and tested in this study, and an exploratory investigation of the role of visual impairment severity has been included. However, future studies should capitalize from joint efforts of multiple laboratories and clinical units to test in a more robust way the impact of associated clinical characteristics (e.g., psychomotor delay) on the sensory–motor integration and attention regulation of infants with visual impairment. Third, the use of gaze orientation as an index of sensory–motor integration might be limited for visually impaired infants [52,53]. This index has largely been used in research on both typically and atypically developing children [54,55,56,57]. Only recently have researchers started looking for valid additional cues of attention direction in infants with typical and atypical developmental trajectories [34].

## 6. Conclusions

This study contributes to the growing body of literature on sensory–motor integration and social attention skills in infants with visual impairment. By examining the influence of maternal caregiving behavior on social attention in sighted and visually impaired infants, our findings have implications for both theoretical understanding and clinical practice. On the one hand, such findings are critical to broaden our comprehension of how the caregiving environment might affect and support socio-cognitive development in infants that present atypical developmental conditions. On the other hand, the present results indirectly support the relevance of delivering early family-centered interventions to empower parents’ role in promoting better outcomes for the development and growth of infants with visual impairment.

## Figures and Tables

**Table 1:** Descriptive statistics and group differences (SIs vs. VI) in maternal measures.

	Sighted Infants		Visual Impairment			
	Mean	SD	Mean	SD	*t (*df*)*	*p*
BDI total score	7.6	7.2	11.0	7.8	−1.41 (36)	0.17
Pragmatic touch (%)	11.2	14.5	11.4	9.5	−0.05 (43)	0.96
Nurturing touch (%)	32.1	15.8	33.7	22.0	−0.27 (43)	0.79
Socio-cognitive verbalization (%)	22.8	15.0	19.0	12.2	0.93 (43)	0.36
Nurturing vocalization (%)	65.1	17.6	66.0	15.7	−0.17 (43)	0.86

Note. BDI—Beck Depression Inventory.

**Table 2:** Correlation matrix (Spearman’s *rho*) for mother–infant measures in the groups.

	Sighted Control (SC) Group			Visual Impairment (VI) Group		
	Unfocused GO	Object GO	Mother GO	Unfocused GO	Object GO	Mother GO
Pragmatic touch (%)	0.464 *	−0.242	−0.103	−0.099	0.453 *	−0.427 *
Nurturing touch (%)	0.191	−0.256	0.005	0.209	0.06	−0.228
Socio-cog. voc. (%)	0.740 ***	−0.028	−0.584 **	−0.306	0.363	−0.114
Nurturing voc. (%)	−0.377	−0.305	0.665 **	0.541 **	−0.599 **	0.329

Note. GO, gaze orientation; * *p* < 0.05, ** *p* < 0.01, *** *p* < 0.001.

## Data Availability

Data are available upon request in an online repository (i.e., Zenodo https://doi.org/10.5281/zenodo.11121406).
